# Rebound hyperkalemia after cessation of ritodrine in a parturient undergoing cesarean section

**DOI:** 10.1186/s40981-016-0071-4

**Published:** 2017-01-05

**Authors:** Daiki Takekawa, Kei Jinushi, Masato Kitayama, Kazuyoshi Hirota

**Affiliations:** 1 Department of Anesthesiology, Hirosaki University Graduate School of Medicine, 5 Zaifu-cho, Hirosaki, 036-8562 Japan; 2 Division of Operating Theater, Hirosaki University Hospital, 53 Hon-cho, Hirosaki, 036-8563 Japan

**Keywords:** Ritodrine, Cesarean section, Hyperkalemia

## Abstract

A 36-year-old parturient with a suspicion of placenta accreta under tocolytic therapy with ritodrine infusion underwent emergency cesarean section under general anesthesia with propofol, ketamine, and remifentanil because massive bleeding was anticipated. The ritodrine infusion was discontinued 1 h before cesarean section. The baby was delivered 6 min after induction of anesthesia. However, after the manual removal of the placenta from the uterus, the bleeding was massive and uncontrollable. We rapidly transfused crystalloid, colloid, and red blood cells through potassium removal filter. Hyperkalemia (5.8 mmol/L) was detected just before blood transfusion. One hour later, hemostasis was still difficult, and hyperkalemia was promoted (6.1 mmol/L). Thus, glucose insulin therapy started with intravenous furosemide to treat hyperkalemia. Gynecologists decided to induce the Bakri balloon tamponade for the treatment of postpartum hemorrhage. At the end of surgery, plasma potassium level also reduced to 5.5 mmol/L. In the ICU, the bleeding still continued, and then radiologists performed bilateral internal iliac artery embolization for full hemostasis. Postoperative plasma potassium level was stable and 3.3 mmol/L in the next morning. Although one of the common adverse reactions of ritodrine is hypokalemia, we should also beware of a rebound hyperkalemia after its cessation.

## Background

Ritodrine, a β_2_-adrenergic agonist, is widely used for tocolytic therapy in parturients. However, one of the common adverse effects of β_2_-agonists is hypokalemia (relative risk, 6.07; 95% confidence interval, 4.00 to 9.20) [1], which is due to an increase in uptake of extracellular potassium by promoting insulin secretion in pancreatic islets with β_2_ adrenoceptor stimulation [2]. However, we experienced a case revealing a rebound hyperkalemia after cessation of ritodrine. This adverse reaction may rarely occur as only five case reports have been found by PubMed search [3–7].

## Case presentation

We have obtained a written informed consent for publication of this case report from the patient.

A 36-year-old parturient (170 cm, 74.8 kg, gravida 1, para 1) with a suspicion of placenta accreta at 34 weeks gestation was admitted into our university hospital. She did not have any abnormal medical history. On the 3rd day after admission, tocolytic therapy against frequent uterine contractions started with intravenous infusion of ritodrine at 50 μg/min. On the 5th day, its infusion dose reduced to 35 μg/min because of her palpitation with tachycardia (HR 120 bpm). On the 6th day, the serum potassium level was 4.1 mmol/L. On the 7th day, as spontaneous amniorrhexis occurred, emergency cesarean section was scheduled, and the ritodrine infusion was discontinued (1 h before cesarean section).

We chose general anesthesia for the emergency cesarean section as massive bleeding due to placenta accreta was anticipated. Anesthesia was induced with intravenous propofol (150 mg) and suxamethonium (50 mg) followed by tracheal intubation uncomplicatedly, and then maintained with propofol (6 mg/kg/h) till delivery. The baby was delivered 6 min after induction of anesthesia. Then, fentanyl and ketamine were additionally given to deepen anesthesia. Rocuronium was also given to maintain muscle relaxation. After the manual removal of the placenta from the uterus, the bleeding was massive and uncontrollable (2200 g in about 20 min). We rapidly transfused colloidal solution against hypotension (63/35 mmHg), and blood pressure increased to 90/50 mmHg within 10 min. We also established an arterial line to left radial artery to maintain blood pressure. Arterial blood gas analyses revealed hyperkalemia (5.8 mmol/L), anemia (Hb 5.0 g/dL), and mild metabolic acidosis (pH 7.33, base excess (BE) −4.5 mEq/L, lactate 1.7 mmol/L). We started blood transfusion with potassium removal filter (potassium adsorption filter (KPF-4), Kawasumi, Tokyo). One hour later, hemostasis was still difficult, and the biochemical date showed further hyperkalemia (6.1 mmol/L) without any electrocardiographic changes and development of metabolic acidosis (pH 7.33, BE –5.2 mEq/L, lactate 1.0 mmol/L). To treat hyperkalemia, glucose insulin therapy started with intravenous furosemide (10 mg). Because of insufficient hemostasis, gynecologists decided to induce the Bakri balloon tamponade for the treatment of postpartum hemorrhage. At the induction of the Bakri balloon, the hematological data were improved (Hb 6.1 g/dL). In addition, plasma potassium level also reduced to 5.5 mmol/L. Thus, the operation was terminated (1 h 54 min) with a total blood loss of 4000 g and urine output of 400 mL. Crystalloid (800 mL), colloid (HES 130/0.4) (1500 mL), 5% albumin (500 mL), and packed red blood cell (6 units) were totally transfused during operation. She postoperatively moved to the intensive care unit under propofol sedation with tracheal intubation. The trachea was extubated after confirming stable hemodynamics and oxygenation. However, as the bleeding still continued, radiologists finally performed bilateral internal iliac artery embolization for full hemostasis. The postoperative course was uneventful and plasma potassium level was 3.3 mmol/L in the next morning. She was discharged from the hospital on foot without any sequelas on the 13th postoperative day.

### Discussion

Ritodrine is a β_2_-mimetic and commonly used for management of preterm labor. The common side effect of ritodrine is hypokalemia as β_2_-adrenoceptor stimulation in pancreatic islets by ritodrine promotes insulin secretion to increase uptake of extracellular potassium [2]. However, in the present case, hyperkalemia occurred 1 h after cessation of ritodrine. Vanishing β_2_-stimulation following cessation of ritodrine conversely increases efflux of potassium from the cells to increase plasma potassium levels. Indeed, there are several similar case reports revealing hyperkalemia following cessation of ritodrine in parturients [3–7]. In addition, in the present case, surgical stress with massive bleeding could reduce insulin secretion and induce insulin resistance by increases in catecholamine and cortisol release. As a result, efflux of potassium from the cell was promoted to enhance hyperkalemia.

In the present case, hyperkalemia became rapidly apparent even 1 h after cessation of ritodrine infusion. It has been reported that plasma ritodrine concentration rapidly declines following termination of its infusion with a distribution half-time of 5.9 ± 6.0 min and a disposition half-time of 156 ± 51 min [8]. Thus, we have to monitor plasma potassium levels around 3 h after cessation of ritodrine. Indeed, Kotani and colleagues [5] reported that maximal hyperkalemia was observed 90–150 min after its cessation in six patients.

Unexpected hyperkalemia following intravenous suxamethonium has also been reported in parturients treated with magnesium and ritodrine under prolonged immobilization [6]. As suxamethonium was used to facilitate tracheal intubation in the present case, it might reinforce ritodrine-induced rebound hyperkalemia. Although plasma potassium levels may increase up to 1.0 mmol/L within 2–5 min following intravenous suxamethonium and the level quickly returns to a baseline value in healthy persons [9], hyperkalemia was detected even 1 h after iv suxamethonium. Therefore, iv suxamethonium may not be the main cause of hyperkalemia in the present case.

The following causes of perioperative hyperkalemia should also be considered: rhabdomyolysis from malpositioning, tissue ischemia, metabolic and respiratory acidosis, and blood transfusion [9]. In the present case, as positioning was carefully done to avoid peripheral nerve injury, rhabdomyolysis could be excluded from the cause. Although the lowest blood pressure was 63/35 mmHg, blood pressure increased to 90/50 mmHg by rapid blood and colloid transfusion. In addition, intraoperative plasma lactate level was also within normal (<2 mmol/L) during surgery. Thus, tissue ischemia could also be excluded. As arterial blood gas analysis revealed mild metabolic acidosis (BE, –4.5 and –5.2 mEq/L), it might cause an increase in plasma potassium level. However, a parturient generally show low arterial CO_2_ with a parallel reduction in plasma HCO_3_^−^ because of hyperventilation induced by effects of progesterone on the respiratory center [10]. Particularly in labor, BE and HCO_3_^−^ decrease to −4.8 and 20.3 mEq/L [10]. Therefore, slight low BE in the present case would be acceptable and would not cause hyperkalemia.

## Conclusions

In summary, we experienced a case of rebound hyperkalemia after cessation of ritodrine in a parturient undergoing an emergency cesarean section with massive bleeding. We should pay attention to perioperative hyperkalemia in a parturient treated with ritodrine for tocolytic therapy.
